# Nasal High-Frequency Oscillatory Ventilation Use in Romanian Neonatal Intensive Care Units—The Results of a Recent Survey

**DOI:** 10.3390/children11070836

**Published:** 2024-07-09

**Authors:** Maria Livia Ognean, Anca Bivoleanu, Manuela Cucerea, Radu Galiș, Ioana Roșca, Monica Surdu, Silvia-Maria Stoicescu, Rangasamy Ramanathan

**Affiliations:** 1 Faculty of Medicine, Lucian Blaga University, 550169 Sibiu, Romania; maria.ognean@ulbsibiu.ro; 2 Neonatology Department, Clinical County Emergency Hospital, 550245 Sibiu, Romania; 3 Regional Neonatal Intensive Care Unit, Grigore T. Popa University of Medicine and Pharmacy, Cuza-Voda Clinical Hospital of Obstetrics and Gynaecology, 700038 Iasi, Romania; 4 Department of Neonatology, George Emil Palade University of Medicine, Pharmacy, Science, and Technology, 540142 Targu Mures, Romania; 5 Department of Neonatology, Emergency County Hospital Bihor, 410167 Oradea, Romania; radu.galis@scjubh.ro; 6 Doctoral School, Poznan University of Medical Sciences, 60-512 Poznan, Poland; 7 Neonatology Department, Panait Sirbu Maternity Hospital, 050786 Bucharest, Romania; 8 Midwifery and Nursing Faculty, Carol Davila University of Medicine and Pharmacy, 020021 Bucharest, Romania; 9 Neonatology Department, County Emergency Hospital Constanța, 900591 Constanta, Romania; monica@surdu.ro; 10 Faculty of Medicine, Ovidius University Constanta, 900470 Constanta, Romania; 11 “Alessandrescu-Rusescu” National Institute for Mother and Child Health, 010919 Bucharest, Romania; 12 Faculty of Medicine, Carol Davila University of Medicine and Pharmacy, 020021 Bucharest, Romania; 13 Division of Neonatal Medicine, Cedars Sinai Guerin Children’s, Cedars Sinai Medical Center, Los Angeles, CA 90048, USA; rangasamy.ramanathan@cshs.org

**Keywords:** nasal high-frequency ventilation, noninvasive ventilation, neonatal intensive care unit, newborn, respiratory distress

## Abstract

Background: Nasal high-frequency oscillatory ventilation (nHFOV) has emerged as an effective initial and rescue noninvasive respiratory support mode for preterm infants with respiratory distress syndrome (RDS); however, little is known about nHFOV use in Romanian neonatal intensive care units (NICUs). Objectives: We aimed to identify the usage extent and clinical application of nHFOV in Romania. Methods: A structured web-based questionnaire was designed to find the rate of nHFOV use and knowledge of this new method of noninvasive respiratory support in Romanian level III NICUs. Using multiple-choice, open-ended, and yes/no questions, we collected information on the NICU’s size, noninvasive respiratory support modes used, nHFOV use, indications, settings, nasal interfaces, secondary effects, and equipment used. Descriptive statistics and comparisons were performed using IBM SPSS Statistics 26.0. Results: A total of 21/23 (91.3%) leaders from level III NICUs (median [IQR] number of beds of 10 [10–17.5]) responded to the survey. The most frequently used noninvasive ventilation modes were CPAP mode on mechanical ventilators (76.2%), followed by NIPPV (76.2%); heated, humidified high-flow nasal cannula (HHHFNC) (61.9%); and nHFOV (11/21 units; 52.4%). A total of 5/11 units reported frequent nHFOV use (in two or more newborns/month) in both term and preterm infants. The main indications reported for nHFOV use were CPAP failure (90.9%), hypercapnia (81.8%), and bronchopulmonary dysplasia (72.7%). Face/nasal masks and short binasal prongs are the most commonly used nasal interfaces (90.9% and 72.7%, respectively). Air leaks at the interface level (90.9%), thick secretions (81.8%), and airway obstruction (63.6%) were the most frequently mentioned adverse effects of nHFOV. Only three of the NICUs had a written protocol for nHFOV use. Most units not yet using nHFOV cited lack of equipment, experience, training, or insufficient information and evidence for the clinical use and outcome of nHFOV use in neonates as the main reasons for not implementing this noninvasive respiratory mode. Conclusions: Our survey showed that nHFOV is already used in more than half of the Romanian level III NICUs to support term and preterm infants with respiratory distress despite a lack of consensus regarding indications and settings during nHFOV.

## 1. Introduction

Despite complex respiratory and hemodynamic changes during the transition to extra-uterine life, most infants adapt smoothly and uneventfully. Many factors may impact the transition to the extra-uterine environment, leading to respiratory distress syndrome (RDS), a condition that occurs particularly in preterm infants. Some infants, both term and preterm, therefore, often require respiratory support at birth or soon after birth. Adequate respiratory support may be life-saving. Invasive mechanical ventilation (IMV) is associated with frequent long-term pulmonary and non-pulmonary morbidities and mortality [1]. Despite continuous increases in the survival of extremely preterm infants, bronchopulmonary dysplasia (BPD) rates are not significantly decreased. Noninvasive ventilation (NIV), defined as any respiratory support not involving the placement of an endotracheal tube, has been shown to reduce the risk of lung injury and associated morbidities [2]. Multiple noninvasive respiratory support modes are currently available, each with its advantages and disadvantages: oxygen administration under the hood or via a low-flow nasal cannula; a heated, humidified, high-flow nasal cannula (HHHFNC); nasal continuous airway positive pressure (NCPAP) with continuous or variable flow; synchronized or non-synchronized nasal intermittent positive pressure ventilation (sNIPPV or NIPPV); nasal bi-level positive airway pressure (BiPAP); nasal high-frequency oscillatory ventilation (nHFOV), nasal high-frequency jet ventilation (nHFJV); and, more recently, noninvasive neurally adjusted ventilatory assist (NIV-NAVA). Choosing the most appropriate NIV mode for each patient and optimizing the NIV support is a continuous pursuit of clinicians working in neonatal intensive care units (NICUs).

Nasal high-frequency oscillatory ventilation (nHFOV), first used in newborns more than 25 years ago [1], is becoming an important initial and rescue noninvasive respiratory support mode for preterm infants with RDS. Following extensive experience with invasive HFOV mode, ventilators with hybrid modes capable of delivering nHFOV mode are now available. Using nHFOV, very small tidal volumes, smaller than the dead space, are delivered to the hypopharyngeal area at supraphysiologic frequency, superimposed on a continuous gas flow during spontaneous respiration, while oscillations are improving alveolar ventilation with an active exhalation phase [3,4,5]. The mechanism of gas exchange, partially similar to invasive HFOV, is still incompletely understood; a synergistic effect of CO_2_ removal at different levels, including in the upper airway dead space, has been proposed [3,4,6]. Oscillation transmission is the most critical variable for gas exchange, but tidal and oscillatory volumes also contribute [4]. Hypothetically, nHFOV should be more efficient than other NIV modes due to its mechanical and biological effects, at least in some clinical situations. Most of the studies performed in recent years confirm the superiority of nHFOV to NCPAP and BiPAP [4,7,8,9,10,11,12] and demonstrate at least the non-inferiority of nHFOV to NIPPV [13,14,15,16,17,18,19] as a primary or rescue NIV mode in preterm infants with RDS or after extubation from IMV. As nHFOV is increasingly recognized as an effective respiratory support mode, its use has increased in Romanian NICUs in recent years. Users who participated in nHFOV training workshops were invited to complete a survey aimed at identifying the usage extent and clinical application of nHFOV in Romania. This paper presents the results of this survey.

## 2. Materials and Methods

### 2.1. Questionnaire

The authors designed a structured web-based questionnaire to find out about the rate of nHFOV usage and knowledge of this relatively new mode of noninvasive respiratory support in Romanian level III NICUs. According to the Romanian legislation on maternal and neonatal care, three levels of care are described. Level III neonatal units provide intensive neonatal care to all preterm infants, irrespective of their gestational age, birth weight, or pathology, compared to level II units that provide care only for infants over 32 weeks’ gestation and with a birth weight over 1500 g. As HFOV and nHFOV are newer respiratory support modes introduced in Romania, the questionnaire was addressed to all level III NICUs in Romania after the units were invited to a workshop on nHFOV but before the workshop took place. The workshop took place in Sibiu between 28 and 29 July 2023.

The questionnaire had four main parts: the first part evaluated the size of the unit by the number of NICU beds in the unit and NICU admissions in the previous year (2022); the second part was composed of questions on the noninvasive respiratory support modes used in the unit; the third and most important part of the questionnaire was addressed to neonatal units using nHFOV and tried to find out how often nHFOV was used, the indications, the initial settings, the minimal and maximal settings during nHFOV, the interfaces used, the secondary effects, and the equipment used to deliver nHFOV; the last part of the questionnaire was addressed to the units not yet using nHFOV, aiming to find out the reasons for not using this mode. The questionnaire had 28 multiple-choice, open-ended, and yes-or-no-answer questions. The questionnaire was sent to the physicians leading the neonatal units participating in the workshop.

### 2.2. Development of the Study

The questionnaire was sent by email and WhatsApp to the leaders of all the level III neonatal units in Romania one day before the workshop began, on 27 July 2023. A reminder was then sent out on 31 July, two days after the workshop ended. The authors monitored the answers to the survey and made a final appeal to those who had not responded via email, WhatsApp, and phone call on 2 August 2023. Of the 23 neonatal units who were invited to answer the questionnaire and participated in the workshop, 21 responded to the survey. All the responses were received by 4 August 2023.

### 2.3. Statistical Analysis

Descriptive statistics was used to analyze reported data. Continuous data are reported as means and standard deviations (SDs) or medians and interquartile ranges (IQRs). Categorical data are reported as numbers and percentages. Comparisons were performed using the Mann–Whitney U test for continuous variables (as they were found to be abnormally distributed) and the chi-square test for categorical variables. A *p*-value < 0.05 was set to describe the statistical significance. Statistical analysis was performed using IBM SPSS Statistics 26.0.

## 3. Results

The survey was completed and returned by 21/23 (91.3%) of the leaders of level III neonatal units in Romania. The number of admissions in the unit during the previous year varied between 250 (reported by a level III NICU functioning in a pediatric hospital, the only such type of NICU in Romania) and 5674 admissions (Table 1). The lowest number of admissions in a maternity hospital reported in the survey was 990. The number of NICU beds in the surveyed neonatal units varied between 8 and 27 (Table 1).

The second part of the survey evaluated the use of noninvasive respiratory support. As seen in Table 2, almost all (95.2%) units use CPAP mode on mechanical ventilators. Nasal intermittent positive pressure ventilation was the second most common NIV mode (76.2%), followed by HHHFNC and bi-level positive airway pressure (BiPAP) (61.9%) and Bubble CPAP (47.6%). Nasal HFOV is used in over half of the surveyed units (52.4%). The preferred interfaces for NIV are short nasal prongs and nasal/facial masks (76.2%), followed by nasal cannulas (61.9%), while facial masks are by far the most used interfaces when NIV is offered in the delivery room (95.2%).

Almost half of the units in the survey reported nHFOV use in two or more patients/month (5/21 units responding to our survey, 23.8%; 5/11 units using nHFOV, 45.5%), while the other units reported rare or occasional nHFOV use (6/11 units using nHFOV, 54.5%) (Table 3). In most units (90.9%), facial/nasal masks are used to deliver nHFOV, followed by short nasal prongs (72.7%). None of the surveyed units reported the exclusive use of nHFOV support in term infants. Seven out of eleven units reported nHFOV use at or soon after birth in term and preterm infants (63.6%) (Table 3).

Continuous positive airway pressure support failure was the most common indication for nHFOV reported in the survey (90.9%), followed by hypercapnia (81.8%) and BPD (72.7%) (Table 3). CPAP levels at which nHFOV is considered as rescue therapy for CPAP failure varied between 6 and 8 cmH_2_O. When switching from CPAP to nHFOV, 10/11 units (90.9%) chose mean airway pressure (MAP) levels equal to the CPAP plus 1–2 cmH_2_O (Table 3).

Various hybrid types of mechanical ventilators are used to deliver nHFOV in 7/11 surveyed units (63.6%) (Table 3). Only 3 of the 11 neonatal units using nHFOV reported an established protocol for this NIV mode. Most of the surveyed units reported leaks at the interface level (90.9%), thick secretions (81.8%), and upper airway obstruction (63.6%) as frequent secondary effects of nHFOV. In contrast, none of the units reported pneumothorax as a complication (Table 3).

Five of the ten units reported a lack of equipment as a reason for not using nHFOV. In comparison, six units reported insufficient experience/training as a reason for not supporting infants with this NIV mode. Insufficient information and evidence for indications, settings, and outcomes were also common (Table 3).

The maximum CPAP level used before switching from CPAP to nHFOV was not significantly different between the units using nHFOV with dedicated equipment (five units) compared to those using hybrid ventilators for delivering nHFOV (seven units) (*p* > 0.05). One NICU reported using dedicated nHFOV equipment and hybrid mechanical ventilators to deliver nHFOV. Also, the initial settings and minimum and maximum frequencies used were similar in the two groups. The initial settings and minimum and maximum amplitudes were significantly higher when using nHFOV on hybrid ventilators (*p* < 0.05). The initial and minimum MAP values were significantly higher with hybrid ventilators (*p* < 0.05). However, the significance level was not reached for the maximum MAP level, although it was still higher than that achieved with non-dedicated equipment (Table 4). The number of admissions and NICU beds during the previous year was similar between the units using dedicated and non-dedicated equipment for nHFOV (Table 4).

We wanted to see if there are any differences between the units rarely/occasionally using nHFOV and those using nHFOV in at least two patients per month (Table 5). We found that more units which frequently support their patients with nHFOV use dedicated equipment (e.g., Medin CNO) (4/5 vs. 1/6) compared to those rarely using this NIV mode. Units frequently using nHFOV reported no machine malfunctioning (0 vs. 2) and no feeding intolerance as secondary outcomes, in contrast to units using this mode in less than one patient per month (Table 5). The equipment used for nHFOV delivery may be related to some of the secondary effects of nHFOV. However, due to the small number of units compared, the statistical significance is limited, and we could not find data in the literature to comparatively analyze these results. Therefore, no definitive conclusion can be drawn.

## 4. Discussion

A systematic review and meta-analysis of individual patient data including 10 trials with 3229 subjects reported information on the adverse effects of invasive HFOV as well as the lack of superiority over conventional mechanical ventilation [20]. In searching for NIV support modes able to reduce mortality and BPD risk in preterm infants with RDS, nasal HFOV emerged as a potentially effective strategy. With the increased availability of hybrid ventilators, able to deliver both invasive (including HFOV support) and NIV, and with more and more studies in the literature, nHFOV has emerged in recent years as an important initial and rescue NIV mode for preterm infants with RDS.

Nasal HFOV combines beneficial effects of CPAP and HFOV: noninvasive nasal interface, increased functional residual capacity, and improved oxygenation offered by CPAP support, and reduced volu-trauma and barotrauma (due to small tidal volumes), optimization of lung volume (determined by almost constant alveolar pressure), increased stability of the distal airways, increased CO_2_ removal, and reduced risk for lung injury offered by HFOV [1,3,21,22]. Also, nHFOV needs no synchronization as it allows spontaneous breathing [5,7,23]. Studies have shown that nHFOV promotes the continuous opening of the glottis same as supraglottic CPAP [24,25], facilitating the interaction between patient and machine [3]. The continuous distending pressure delivered during NHFOV significantly reduces the shear stress of the airways and lungs, potentially reducing the BPD risk [3]. Another important advantage of nHFOV is the possibility of using different interfaces: nasal/facial masks, short nasal prongs, RAM nasal cannula, and nasopharyngeal tubes. Oscillations in nHFOV significantly reduce the surface tension, possibly improving surfactant function [26]. Studies in animal models showed the increased production of surfactant protein B and better alveolarization [25,27]. Based on these effects, de Luca and Dell’ Orto [3] speculated that oscillations applied before surfactant administration may improve surfactant function. Rehan et al. [27], in a study on an animal model of BPD, showed that nHFOV promotes the coordinated expression of epithelial and mesenchymal paracrine signaling pathways of alveolar homeostasis compared to the inhibiting effect of IMV. A lack of paracrine signaling and the immaturity of alveolar type II cells are associated with lesions secondary to barotrauma, hyperoxia, and infection, which impact cellular differentiation and increase the BPD risk [27]. Nasal HFOV was associated with reduced inflammatory markers compared to conventional ventilation [28]. Studies with electrical impedance tomography have shown that oscillatory volumes are substantially transmitted to the lungs, including in areas non-dependent on gravity during nHFOV [29], and there is increased and more homogeneous aeration, increased end-expiratory lung volumes, and similar homogeneity of ventilation as compared to NCPAP [30]. Hypothetically, nHFOV should be more efficient than other NIV modes due to all these effects, at least in some particular clinical situations. Most of the studies performed in recent years confirm the superiority of nHFOV to NCPAP and BiPAP [5,7,8,9,10,11,12] and demonstrate at least the non-inferiority of nHFOV to NIPPV [13,14,15,16,17,18,19] as a primary or rescue NIV support in preterm infants with RDS or after extubation from IMV.

Our study is the first performed in Romania regarding the utilization and clinical application of nHFOV. As more of the literature supports this novel mode of NIV support, experts worldwide are trying to more clearly establish guidelines for nHFOV regarding indications, appropriate settings, and weaning from nHFOV. More information and training are needed for Romanian neonatologists. In Romania, according to our legislation regarding the regionalization of maternal and neonatal care [31,32], level III neonatal units offer the highest level of neonatal care.

The response rate to our survey was similar (91.2 versus 92%) to that of Fischer et al. [33] in 2015 from five European countries regarding the use of nHFOV. In our study, the clinical experience with nHFOV was markedly higher than in the study of Fischer et al. [33], which reported that 30 of the 172 units answering the survey (17%) were using nHFOV (5% in Sweden to 20% in Germany). Low nHFOV rates of 18% were also reported in Canada by Mukerji et al. [34] in 2016, based on a survey of 28 neonatal centers. Another survey, developed in Italy in 2019 and involving 113 level III neonatal centers, has shown that nHFOV was used only in 6/113 centers (5.3%) [35]. We have not found recent data to compare the nHFOV usage rate in our survey. Interestingly, another Romanian survey recently reported that in 2018, invasive HFOV was used in 60.6% of the level II and III Romanian NICUs (20 of 33 units responding to the survey) [36], suggesting an increased interest and experience with HFOV and possibly nHFOV (with the increased number of hybrid ventilators) in Romania.

Our survey also included questions about NIV modes used in the level III NICUs. We have found that nasal CPAP, either with specific equipment or using a mechanical ventilator, was the most used NIV mode in the NICUs (over 95.2% and 66.6%, respectively), similar to 100% CPAP use reported by Petrillo et al. [35], Mukerji et al. [34], and Cucerea et al. [36]. The rate of BiPAP use was lower (47.6%) compared to the 79% rate reported by Mukerji et al. [34] and 74.3% reported by Petrillo et al. [35]. We were surprised by the increased use of NIPPV in Romania (76.2%) as compared to the 54% rate reported in Canada [34] and 40% reported in Italy [35]. However, the explanation may be that these surveys dated back to more than five years ago, and NIPPV has emerged as an important rescue NIV mode in recent years [15,18].

The preferred interfaces used for NIV in our survey were short nasal prongs, facial/nasal masks (both used in 76.2% of the responding centers), and nasal cannulas (61.9%), with rates comparable with those reported by Fischer et al. [33] (73% for short nasal prongs). Nasopharyngeal tubes (9.5%) and RAM cannulas (23.8%) are rarely used in Romanian NICUs for delivering NIV, and even more rarely in the delivery room (none of the units reported use of nasopharyngeal tubes for NIV in the delivery room, while only one unit reported use of RAM cannulas). This information contrasts the frequency of 63% use of a single nasopharyngeal tube for NIV reported by Fischer et al. [33]. The high usage rate of masks in the delivery room in our survey (95.2%) is higher than the rate reported by Gizzi et al. [37] for centers caring for high-risk neonates (83.6%); short nasal prongs were more often used in level III units in Romania in the delivery room compared to similar units in Italy (23.8% versus 11.8%), while the nasopharyngeal tube was also rarely used in Italian centers. In vitro mechanical studies performed by de Luca et al. [38] have shown that nHFOV is feasible using common nasal prongs but its efficiency depends on prongs’ diameter and stiffness; however, efficient tidal volume can be ensured by changes in the amplitude and inspiratory time even if using small-sized prongs [39]. A recent European survey [40] found significantly different preferences for NIV interfaces in the delivery room, but face masks were also the most used (75.9%); short binasal prong use was reported with rates similar to our survey (20.2%), while nasopharyngeal tubes (either binasal or single) were rarely reported (less than 4%). The recent Romanian report on neonatal resuscitation and stabilization practices in the delivery room confirms that the face mask is the most frequently used interface in level III neonatal units (72.7%), followed by the short binasal prongs (27.3%) [36].

As the new guidelines for RDS management are recommending NIV and less invasive surfactant administration in preterm infants with spontaneous breathing and clinical signs of RDS [41], we were also curious about the frequency of use of less invasive surfactant administration (LISA) and intubation versus surfactant administration followed by extubation on NIV support (INSURE) techniques. The survey showed a clear trend for these approaches compared to the older data [36]—LISA use increased from 7.1% to 66.7%, and INSURE use increased from 40.0% to 71.4%, suggesting an increased interest in reducing the risk of intubation and prolonged mechanical ventilation and good compliance with European consensus guideline recommendations.

Nasal HFOV is used quite frequently in almost half of the level III units in our survey; still, a protocol for nHFOV use is in place in only three of these units. According to the answers received, nHFOV is used in all categories of preterm infants and in term infants in most of the surveyed units (Table 3). Also, Romanian neonatal units use nHFOV for all the indications reported in the literature, most commonly as a rescue NIV after CPAP failure (90.9%), similar to the results of Fischer et al.’s [33] study that reported nHFOV use for CPAP failure in 90% of the units in their survey, primarily in preterm infants with birth weight <1500 g with CPAP failure. Interestingly, the reported CPAP levels considered for switching from CPAP to nHFOV are pretty low (6–7 cmH_2_O reported by 7 of the 11 units using nHFOV, while the other 4 units reported 8 cmH_2_O. A minimum CPAP level of 6 cmH_2_O is recommended in the European guidelines for RDS management [41]. Initial CPAP levels of 5–7 cmH_2_O were reported in the 2018 survey in preterm infants with RDS in the delivery room by all 12 level III neonatal units in Romania [36]. Nasal HFOV support was reported as successful as a rescue mode starting in 1998 by Hoeven et al. [42]; 77% of the patients in the study, term and preterm infants with moderate respiratory failure, avoided IMV. Another study retrospectively evaluated the efficiency of nHFOV as rescue therapy after CPAP or BiPaP support and found a success rate of 69% in avoiding intubation [43]. No difference was noted in our survey regarding the means and median (IQR) values of CPAP level before switching from CPAP to nHFOV (*p* = 0.931) (Table 4).

A high percentage (81.8%) of the surveyed units reported nHFOV use in patients with hypercapnia, far more frequent compared to Fischer et al.’s survey [33], most probably due to numerous studies that reported improved CO_2_ removal with this NIV mode compared to CPAP or NIPPV [4,44,45,46,47,48]. Eight of eleven units in our survey reported nHFOV use as a respiratory support mode in BPD. This indication is still unclear in the literature. However, as BPD may be considered a mixed model of restrictive and obstructive respiratory disease characterized by hypercapnia and hypoxemia, it may benefit from the effects of nHFOV [3]. Nasal HFOV may be helpful in the evolving, exudative phase of BPD, a situation characterized by increased resistance of the airways and alveolar edema with decreased compliance as nHFOV allows the use of higher mean airway pressure, avoiding air trapping and improving hypercarbia [4,49]. De Luca and Dell’Orto [3] suggested starting settings for nHFOV in patients with evolving BPD.

The most studied indication for nHFOV support is after extubation from IMV. Nasal HFOV is used as respiratory support after extubation in 7 of the 11 units in our survey, similar to the results of another report (15 of the 30 units) [33]. Recent studies, systematic reviews, and meta-analyses support nHFOV as an efficient NIV strategy post extubation in preterm infants. Compared to NCPAP [7,14,16,47,50] and BiPaP [4], nHFOV was associated with significantly lower rates of reintubation and more effective CO_2_ removal. Compared to NIPPV [13,14,15,16,19], nHFOV significantly reduced reintubation rates, was more efficient in CO_2_ removal, and decreased IMV duration. In another recent randomized controlled trial including extremely preterm infants, nHFOV and NIPPV, compared to NCAP, significantly reduced the reintubation rate, with a number needed to treat of 3 to 7, and nHFOV significantly decreased the moderate to severe BPD rate, with a number needed to treat of 8–9 [17]. A significantly reduced rate of reintubation is clinically significant as reintubation is associated with an increased risk of BPD and death [51]. Therefore, nHFOV may provide an important option in choosing the NIV support mode after extubation in preterm infants. A recent report suggests that using nHFOV after extubation significantly reduces BPD rates and length of hospitalization [16].

Many studies in the literature support nHFOV use as primary NIV support in preterm infants with RDS, an indication also suggested by the most recent European guideline on RDS management [41]. In our survey, six units (54.5%) reported using nHFOV as initial respiratory support in RDS and for alveolar recruitment, suggesting compliance with the most recent recommendations and knowledge of the results of the studies in the literature. The first RCT comparing nHFOV with NCPAP as the primary support mode in preterm infants born at 28–34 weeks’ gestation showed significantly lower rates of mechanical ventilation with nHFOV (*p* < 0.01) and decreased BPD and mortality rates (*p* > 0.5) using non-dedicated equipment for nHFOV delivery [52]. A significantly reduced need for intubation was reported by a large study in China with nHFOV as the primary support mode in preterm infants with RDS compared to NCPAP (*p* = 0.016) and NIPPV (*p* = 0.027) [13]. Significantly decreased rates of intubation and BPD risk were also reported by another RCT performed in preterm infants for the entire study group (N = 88) and in subgroups based on gestational age (26–29 weeks and 30–32 weeks of gestation, respectively) [53]. With nHFOV as the primary support mode in preterm infants with RDS, the reduced risk of intubation was also reported in a recent meta-analysis compared to NCPAP—relative risk (RR): 0.47 (95% CI 0.30–0.70); *p* = 0.0002 [9]; RR 0.45 (95% CI: 0.37–0.55); *p* < 0.01 [12]—and compared to CPAP and BiPAP—RR 0.50 (95% CI: 0.36–0.70) [9]. Unfortunately, experts are still searching for the best candidates and the most suitable clinical indications for nHFOV to improve the outcomes of NICU patients [5,54,55].

Our survey, similar to Fisher et al.’s [33], found that nHFOV is delivered in the NICU with various types of ventilators specifically designed for nHFOV (e.g., Medin CNO) or hybrid mechanical ventilators (Table 3). The choice of equipment is likely influenced by each unit’s financial resources, experience, and preference, highlighting the practical considerations in nHFOV delivery.

De Luca et al. [3,56] suggested the face mask as the most efficient interface for oscillation transmission and tidal volume delivery with nHFOV as oscillation attenuation is reduced compared to other interfaces. The same was demonstrated by King et al. [57]. Our survey showed that face/nasal masks are the preferred interfaces for nHFOV delivery by 10 of the 11 units surveyed. Short nasal prongs are also used extensively, with eight units indicating their use with nHFOV. Our results are in contrast to the survey performed by Fischer et al. [33] that showed a preference for nasal prongs (22 of 30 NICUs) and a single nasopharyngeal tube (19 NICUs) and a low rate of face/nasal masks (8 NICUs). The Canadian survey [34] showed similar results: a preference for nasal masks and short binasal prongs with nHFOV use.

The interpretation of the answers to questions on parameters used when initiating and during nHFOV is quite difficult, as dedicated ventilators and hybrid ventilators use different settings to deliver nHFOV. Also, in clinical practice, starting values of the frequency, amplitude, and mean airway pressure also depend on the clinical indication and clinical condition of the patient. This is one problem encountered by experts who tried to define recommendations for nHFOV settings with different equipment and in different situations (e.g., post extubation, in BPD) [3,10,58]. Some studies recommend setting a MAP equal to or higher than 1–2 cmH_2_O compared to CPAP or PEEP when using nHFOV as a rescue mode from other NIV respiratory support [43,49,59] or adjusting MAP, such as during recruitment maneuvers [7]. The initial, minimum, and maximum frequency, amplitude, and MAP values reported in our survey were compared between units using dedicated equipment for nHFOV support (Medin CNO) and those using mechanical ventilators (Table 4). We have found no difference in the frequencies used with nHFOV between the two study groups. As expected, the amplitudes used with dedicated nHFOV ventilators were significantly lower than those used with non-dedicated ventilators (*p* < 0.017). The MAP values were also lower with special nHFOV machines, with the difference being significant only for initial and minimum MAP values (*p* = 0.004). Compared to the results of the survey published by Fischer et al. [33] that used mostly nHFOV delivered by mechanical ventilators, our survey showed similar median frequencies (10 Hz) and oscillation amplitudes (20 cmH_2_O) and higher MAP values (10 cmH_2_O vs. 8 cmH_2_O). The survey published by Mukerji et al. [34] does not specify the type of ventilators used for nHFOV. Therefore, comparing the parameters used in the surveys would be difficult. No difference was observed between the units with regard to the number of NICU beds and admissions during the year prior to the survey (Table 4). However, bigger units tend to use dedicated ventilators more frequently to provide nHFOV.

Most studies published in the literature report that nHFOV is a safe NIV mode [4,60], with beneficial results and no increase in adverse effects [7,13,48,52,61]. Analysis of the part of our survey evaluating the secondary effects of nHFOV showed that leaks at the interface level (90.9%), thick secretions (81.8%), and secondary obstruction of the upper airway (63.9%) were the most common adverse effects noted by clinicians (Table 3). This is in contrast to the results reported by Fischer et al. [33], which showed that abdominal distension was the most common side effect observed (37% compared to 45.5% in our survey), followed by secretions obstructing the upper airways and thick secretion but with lower rates than our survey (27% and 23%, respectively). The increased leak rate at the interface level in our survey may be explained by the type of interface used—face/nasal masks in our survey—compared to prongs used by units reported in Fischer et al.’s study [33]. Increased secretions and secondary airway obstruction are often reported in the literature as side effects of nHFOV [18,33] and are attributed to insufficient or inadequate gas conditioning as some of the humidifiers are not reaching the expected temperature and humidity [62,63,64]. A combination of low frequencies, high amplitudes, and increased inspiratory to expiratory time significantly reduce the temperature and absolute humidity of the gasses, increasing the risk for dryness of the upper airways [62]. Agitation was noted far more often than in Fischer et al.’s study [33] (38.1% vs. 3%); this may have been due to the patient’s discomfort with the interface or ineffective nursing strategies. Intolerance to feedings was reported by three of the units using nHFOV; others did not mention this side effect, and one comparative study mentioned decreased feeding tolerance with NIPPV compared to nHFOV [1]. One study suggested that nHFOV, similar to NCPAP and NIPPIV, inhibits gastro-esophageal reflux in newborn lambs, mentioning that abdominal distension is the main trigger for inferior esophageal sphincter relaxation and reflux occurrence [65].

Interest in nHFOV use among Romanian neonatologists is increasing. Lack of equipment was reported by 5 of the 10 units not using this nHFOV as the main reason for not using this NIV respiratory support, and 6 units reported insufficient nHFOV experience or training (Table 4). Insufficient information or evidence on indications, settings, and outcomes were also reported in 30–40% of the cases. Although information and evidence on the beneficial effects and advantages of nHFOV are continuously increasing, experts are reporting that we still need more clear clinical indications for nHFOV (gestational age or birth weight criteria, types of respiratory conditions, severity of respiratory failure, settings for different indications) [4,15,43]. Further studies may help establish a hierarchy of NIV support, with a more precise position of nHFOV among other NIV modes. There is also an urgent need for clear criteria to define nHFOV failure. As regards the information on the outcomes of the infants supported using nHFOV, up to now, there have been few studies suggesting that similar to HFOV, there is no adverse effect on cerebral hemodynamics [66] and no adverse effects compared to NCPAP and NIPPV after extubation [67]. Indeed, we need more studies in this respect.

Our study has limitations that must be acknowledged. The number of units participating in the questionnaire is low, but over 90% of the units involved in the care of high-risk neonates in Romania responded; thus, we consider the results to be representative of nHFOV use, a recently introduced respiratory support mode, in the country. There are aspects of nHFOV usage in practice not covered by the questionnaire, such as the inspiratory to expiratory ratio. The survey was designed for rapid completion. Thus, some questions related to parameter settings had multiple-choice answers, not allowing open answers, for example, other ways to choose the starting MAP on nHFOV or multiple choices for parameter settings with different indications of nHFOV. The timing of the survey around the training workshop on nHFOV could have been a source of bias. However, the survey was addressed to the leaders of the level III neonatal units who did not participate in the workshop. The statistical power of the comparative statistics between NICUs using nHFOV with dedicated versus hybrid ventilators must be interpreted with caution. Our survey did not evaluate the success rate of nHFOV. Still, the survey demonstrates an increased awareness and knowledge of nHFOV and an interest in extending its use in clinical practice. Also, the survey shows compliance with recent recommendations of the European guidelines for RDS management in preterm infants.

## 5. Conclusions

The survey on the extent of nHFOV usage and the clinical experience with nHFOV in more than 90% of the level III neonatal units in Romania in 2023 showed that nHFOV is already used in more than half of these units on a regular basis. Attempts are made to avoid IMV through a noninvasive approach to respiratory support and surfactant administration. As no definitive categories of newborns may benefit from nHFOV support and nHFOV indications still need to be defined, each unit should develop its own protocol, considering the equipment used for nHFOV delivery. Multicentric studies are also needed to evaluate the impact of nHFOV in similar circumstances on the most important outcomes, particularly in extremely preterm infants: BPD, death, and neurodevelopmental outcomes. As a need for training for nHFOV use was identified, efforts are needed at the national level to develop courses and training sessions, including using simulations, to offer more information on nHFOV use in clinical practice.

## Figures and Tables

**Table 1:** Characteristics of the surveyed neonatal units.

	Mean ± SD ^2^	Range	Median (IQR ^3^)
Number of admissions/year 2022	2648.5 ± 1352.8	250–5674	2350 (1550–3466)
NICU ^1^ beds	13.6 ± 6.3	8–27	10 (10–17.5)

Legend: ^1^ NICU—neonatal intensive care unit, ^2^ SD—standard deviation, ^3^ IQR—interquartile range.

**Table 2:** Noninvasive respiratory support.

	N ^8^ (%)
Types of noninvasive respiratory support	
- CPAP ^1^ on mechanical ventilators	20/21 (95.2)
- nCPAP ^1^	4/21 (19.0)
- Bubble CPAP ^1^	10/21 (47.6)
- BiPAP ^2^	10/21 (47.6)
- HHHFNC ^3^	13/21 (61.9)
- NIPPV ^4^	16/21 (76.2)
- nHFOV ^5^	11/21 (52.4)
Interfaces on noninvasive respiratory support	
- short binasal prongs	16/21 (76.2)
- facial/nasal mask	16/21 (76.2)
- RAM cannula	5/21 (23.8)
- nasopharyngeal tube	2/21 (9.5)
- nasal cannula	13/21 (61.9)
Preferred interfaces in the delivery room	
- facial mask	20/21 (95.2)
- short nasal prongs	5/21 (23.8)
- RAM nasal cannula	1/21 (4.8)
The preferred method for surfactant administration in preterm infants spontaneously breathing	
- INSURE ^6^	15/21 (71.4)
- LISA ^7^	14/21 (66.7)

Legend: ^1^ CPAP—continuous positive airway pressure, ^2^ BiPAP—bi-level positive airway pressure, ^3^ HHHFNC—heated, humidified, high-flow nasal cannula, ^4^ NIPPV—nasal intermittent positive pressure ventilation, ^5^ nHFOV—nasal high-frequency oscillatory ventilation, ^6^ INSURE—intubatio–-surfactant–extubation; ^7^ LISA—less invasive surfactant administration, ^8^ N—number.

**Table 3:** Data reported on nasal high-frequency oscillatory ventilation (nHFOV) use.

	N ^7^ (%)
Frequency of nHFOV use	
- rare (<1 patient/2 months)	4/21 (19.0)
- occasional (1 patient/month)	2/21 (9.5)
- frequent (2 patients/month)	3/21 (14.3)
- often (>2 patients/month)	2/21 (9.5)
- never	10/21 (47.7)
Categories of patients in which nHFOV is used	
- <28 weeks GA ^1^	8/11 (72.7)
- <32 weeks GA ^1^	7/11 (63.6)
- <1000 g BW ^2^	8/11 (72.7)
- <1500 g BW ^2^	8/11 (72.7)
- all preterm infants	7/11 (63.6)
- term neonates	0
- preterm and term neonates	7/11 (63.6)
Indications for nHFOV	
- initial respiratory support in RDS ^3^	6/11 (54.5)
- alveolar recruitment	6/11 (54.5)
- CPAP ^4^ failure	10/11 (90.9)
- post extubation	7/11 (63.6)
- hypercapnia	9/11 (81.8)
- BPD ^5^	8/11 (72.7)
Maximum CPAP level before switching to nHFOV	
- 5 cmH_2_O	0
- 6 cmH_2_O	6/11 (54.5)
- 7 cmH_2_O	1/11 (9.1)
- 8 cmH_2_O	4/11 (36.4)
- 9 cmH_2_O	0
- >9 cmH_2_O	0
MAP ^6^ compared to CPAP levels when switching from CPAP to nHFOV	
- MAP = CPAP	2/11 (18.2)
- MAP < CPAP	0
- MAP = CPAP + 1–2 cmH_2_O	8/11 (72.7)
- MAP > CPAP + 2 cmH_2_O	2/11 (18.2)
Type of equipment used for nHFOV	
- dedicated machines (e.g., Medin CNO)	5/11 (45.5)
- mechanical ventilators	7/11 (63.6)
Equipment used for nHFOV	
- Draeger	1/11 (9.1)
- Fabian	4/11 (36.4)
- Sensormedics	1/11 (9.1)
- Leoni	4/11 (36.4)
- SLE	4/11 (36.4)
Interfaces used for nHFOV	
- short nasal prongs	8/11 (72.7)
- face/nasal mask	10/11 (90.9)
- RAM cannula	1/11 (9.1)
- nasopharyngeal tube	1/11 (9.1)
Secondary effects observed on nHFOV	
- abdominal distension	5/11 (45.5)
- upper airway obstruction	7/11 (63.6)
- thick secretions	9/11 (81.8)
- intolerance/altered feeding tolerance	3/11 (14.3)
- agitation	8/11 (38.1)
- pneumothorax	0
- leaks at the interface	10/11 (90.9)
- equipment malfunction	2/11 (18.2)
Existence of an nHFOV protocol	3/11 (27.3)
Reason for not using nHFOV	
- lack of equipment	5/10 (50.0)
- insufficient information on indications	4/10 (40.0)
- insufficient information on settings	4/10 (40.0)
- insufficient information on outcomes	4/10 (40.0)
- insufficient evidence for indications	3/10 (30.0)
- insufficient evidence for settings	3/10 (30.0)
- insufficient evidence for outcomes	3/10 (30.0)
- insufficient experience/training	6/10 (60.0)

Legend: ^1^ GA—gestational age, ^2^ BW—birth weight, ^3^ RDS—respiratory distress syndrome, ^4^ CPAP—continuous positive airway pressure, ^5^ BPD—bronchopulmonary dysplasia, ^6^ MAP—mean airway pressure, ^7^ N—number.

**Table 4:** Nasal high-frequency oscillatory ventilation (nHFOV) settings—comparison between nHFOV with dedicated equipment and nHFOV with mechanical ventilators.

	nHFOV with Dedicated Equipment (5 NICUs ^3^)		nHFOV with Mechanical Ventilators (7 NICUs ^3^)		*p*
	Mean ± SD ^4^	Median (IQR ^5^)	Mean ± SD ^4^	Median (IQR ^5^)	
Maximum CPAP ^1^ before switching to nHFOV (cmH_2_O)	6.8 ± 1.1	6 (6–8)	6.8 ± 1.0	6.5 (6–8)	0.931
Frequency (Hz)					
- initial	10.2 ± 1.1	10 (9.50–11)	10.2 ± 0.4	10 (10.0–10.25)	0.792
- minimum	8.8 ± 1.1	8 (8.0–10.0)	8.5 ± 1.5	8.5 (7.5–10.0)	1.000
- maximum	14.2 ± 1.3	15 (13.0–15.0)	14.3 ± 1.7	14.5 (12.75–15.50)	1.000
Amplitude (cmH_2_O)					
- initial	11.6 ± 4.8	10 (9.0–15.0)	21.3 ± 5.3	20 (18.5–25.5)	0.017
- minimum	8.8 ± 4.0	8 (5.5–12.5)	16.3 ± 3.1	15.5 (14.25–20.0)	0.017
- maximum	13.8 ± 6.5	10 (10.0–19.5)	31.0 ± 7.8	30 (24.5–40.0)	0.009
MAP ^2^ (cmH_2_O)					
- initial	6.2 ± 1.1	6 (5.5–7)	12.7 ± 5.0	10 (9.5–18.5)	0.004
- minimum	5.6 ± 0.5	6 (5–6)	10.8 ± 3.7	9 (8–15.25)	0.004
- maximum	12.0 ± 4.1	15 (7.5–15.0)	20.5 ± 8.4	18 (14.25–27.5)	0.052
No of NICU ^3^ beds	16.4 ± 7.9	12 (10.0–25.0)	13.2 ± 7.4	10.5 (8–18)	0.537
No of admissions/year 2022	3721.4 ± 1517.2	3300 (2406–5247)	2006.5 ± 1535.5	1550 (805–3610)	0.126

Legend: ^1^ CPAP—continuous positive airway pressure, ^2^ MAP—mean airway pressure, ^3^ NICU—neonatal intensive care unit, ^4^ SD—standard deviation, ^5^ IQR—interquartile range.

**Table 5:** Comparison of nasal frequency oscillatory ventilation (nHFOV) use between units reporting frequent versus rare usage of this noninvasive respiratory support mode.

	Units Rarely Using nHFOV (N ^2^ = 6)	Units Frequently Using nHFOV (N ^2^ = 5)	*p*
Number of beds (mean ± SD ^1^)	12.8 ± 6.7	16.8 ± 8.5	0.407
Number of deliveries in 2022 (mean ± SD)	2910.5 ± 1857.5	2636.6 ± 1700.1	0.806
Categories of patients in which nHFOV is used (N ^2^/%)			
- <28 weeks GA ^3^	5/83.3	3/60.0	0.439
- <32 weeks GA ^3^	4/66.7	3/60.0	0.840
- <1000 g BW ^4^	5/83.3	3/60.0	0.438
- <1500 g BW ^4^	5/83.3	3/60.0	0.438
- all preterm infants	4/66.7	3/60.0	0.840
- term neonates	4/66.7	3/60.0	0.840
Indications for nHFOV (N/%)			
- initial respiratory support in RDS ^5^	2/33.3	3/60.0	0.148
- alveolar recruitment	3/50.0	4/80.0	0.770
- CPAP ^6^ failure	5/83.3	5/100	0.389
- post extubation	4/663.7	3/60.0	0.840
- hypercapnia	4/66.7	5/100	0.186
- BPD ^7^	4/66.7	4/80.0	0.662
Maximum CPAP ^6^ level before switching to nHFOV (mean ± SD ^1^)	6.7 ± 1.0	7.0 ± 1.0	0.333
Use of dedicated nHFOV machines (e.g., Medin CNO) (N ^2^/%)	1/16.7	4/80	0.067
Secondary effects reported on nHFOV (N ^2^/%)			
- abdominal distension	3/50.0	2/40.0	0.770
- upper airway obstruction	3/50.0	4/80.0	0.353
- thick secretions	5/83.3	4/80.0	0.900
- intolerance/altered feeding tolerance	3/50.0	0	0.074
- agitation	5/83.3	3/60.0	0.438
- pneumothorax	0	0	-
- leaks at the interface	5/83.3	5/100	0.389
- equipment malfunction	2/33.3	0	0.186
Existence of an nHFOV protocol	2/33.3	1/20.0	0.662

Legend: ^1^ SD—standard deviation, ^2^ N—number, ^3^ GA—gestational age, ^4^ BW—birth weight, ^5^ RDS—respiratory distress syndrome, ^6^ CPAP—continuous positive airway pressure, ^7^ BPD—bronchopulmonary dysplasia.

## Data Availability

All the data presented in the study are included in the article; further inquiries can be directed to the corresponding author(s). The questionnaire used in the study is offered as Supplementary Material.

## Supplementary Materials

The following supporting information can be downloaded at: https://www.mdpi.com/article/10.3390/children11070836/s1, File S1. Survey on non-invasive high frequency respiratory support.
