# Supplementary material for: Nasal High-Frequency Oscillatory Ventilation Use in Romanian Neonatal Intensive Care Units—The Results of a Recent Survey
Source: Children (Basel). 2024 Jul 9;11(7):836. doi: 10.3390/children11070836 (PMC11276281; doi:10.3390/children11070836)
Supplement: Supplementary file 1 [file children-11-00836-s001.zip › children-3069945-supplementary.pdf]

Survey on non-invasive high frequency respiratory support

Section 1

1. Location
2. Level
3. Number of births in 2022
4. What kind of non-invasive respiratory (NIV) modes are you using in your unit for preterm infants, please choose all that apply:
    a. Ventilator CPAP
    b. Bubble CPAP
    c. BiPAP
    d. NIPPV
    e. NHFOV
    f. HFNC
    g. Others, please specify
5. Which interface is preferred in your unit when using non-invasive ventilation (NIV):
    a. Short binasal prongs
    b. Face/Nasal mask
    c. RAM cannula
    d. Nasopharyngeal tube
6. Which interface is preferred during resuscitation in the delivery room?
    a. Face mask
    b. Short binasal prongs
    c. Ram cannula
7. In preterm infants needing NIV respiratory support and surfactant administration how is surfactant administered:
    a. INSURE
    b. LISA/MIST

Section 2: If you are using non-invasive high frequency oscillation ventilation (NHFOV)

1. How often do you use NHFOV support>
    a. Rarely (less than 1 patients in 2 months)
    b. Occasionally (1 patient/month)
    c. Frequently (2 patients/month)
    d. Often (more than 2 patients/month)
2. What type of equipment you are using for NHFOV support:
    a. Dedicated equipment for NHFOV (eg. Medin CNO)
    b. Ventilators with NHFOV mode
3. Is you are using a ventilator for NHFOV delivery, please chose the ones you are using in your unit:
    a. Draeger
    b. Fabian
    c. Sensormedics
    d. Leoni
    e. Other, please specify
4. Which is the preferred interface on NHFOV in your unit:
    a. Short binasal prongs
    b. Face/nasal mask
    c. Ram cannula
    d. Nasopharyngeal tube
5. Which of the following are used as indications for NHFOV in your unit, please choose all that apply:

    a. Initial respiratory support in preterm infants with RDS
    b. Alveolar recruitment in preterm infants with RDS
    c. CPAP failure
    d. Postextubation
    e. Hypercapnia
    f. Evolving BPD/BPD
6. If you are using NHFOV as escalation strategy, which is the maximum PEEP or CPAP before initiating NHFOV:
    a. 5 cmH2O
    b. 6 cmH2O
    c. 7 cmH2O
    d. 8 cmH2O
    e. 9 cmH2O
    f. Over 9 cmH2O
7. Please note the start value of frequency when using NHFOV:

8. Please note the minimum value of frequency when using NHFOV:

9. Please note the maximum value of frequency when using NHFOV:

10. Please note the start value of amplitude when using NHFOV:

11. Please note the minimum value of amplitude when using NHFOV:

12. Please note the maximum value of amplitude when using NHFOV:

13. Please note the start value of mean airway pressure (MAP) when using NHFOV:

14. Please note the minimum value of mean airway pressure (MAP) when using NHFOV:

15. Please note the maximum value of mean airway pressure (MAP) when using NHFOV:

16. If you are switching from CPAP to NHFOV, how is MAP on NHFOV set:
    a. Similar
    b. Lower
    c. Higher by 1-2 cmH2O
    d. Higher by more than 2 cm H2O
17. Are you using NHFOV in (please, choose all that apply):
    a. Preterm infants under 28 weeks gestation
    b. Preterm infants under 32 weeks gestation
    c. Preterm infants under 1000 g
    d. Preterm infants under 1500 g
    e. All kind of preterm infants
    f. Term and preterm infants
    g. Only term neonates
18. Please, choose what kind of secondary effects you have seen in patients on NHFOV:
    a. Abdominal distension

    b. Upper airway obstruction
    c. Thick secretions
    d. Problems with feeding tolerance
    e. Agitation, irritability
    f. Pneumothorax
    g. Leaks at the interface
    h. Equipment malfunction
    i. Others, please specify:

19. Do you have a written protocol for NHFOV use in neonates?
    a. Yes
    b. No
20. If you are using NHFOV, please state what are the areas you need more information (open answer):

21. If you are not using NHFOV in your unit, please state why:
    a. We do not have the equipment needed (ventilators with NHFOV, dedicated machine for NHFOV)
    b. Insufficient information regarding:
        i. Indications
        ii. Settings
        iii. Outcomes
    c. Insufficient evidence regarding:
        i. Indications
        ii. Settings
        iii. Outcomes
    d. Insufficient experience/training
